# Laparoscopic surgery for diverticular colovesical fistula: single-center experience of 11 cases

**DOI:** 10.1186/s13104-020-05022-4

**Published:** 2020-03-24

**Authors:** Daichi Kitaguchi, Tsuyoshi Enomoto, Yusuke Ohara, Yohei Owada, Katsuji Hisakura, Yoshimasa Akashi, Kazuhiro Takahashi, Koichi Ogawa, Osamu Shimomura, Tatsuya Oda

**Affiliations:** Department of Gastrointestinal and Hepato-Biliary-Pancreatic Surgery, Faculty of Medicine, University of Tsukuba, 1-1-1 Tennodai, Tsukuba, Ibaraki 305-8575 Japan

**Keywords:** Colovesical fistula, Diverticular fistula, Laparoscopic surgery, Conversion to open surgery

## Abstract

**Objective:**

Laparoscopic surgery for diverticular colovesical fistula (CVF) is technically challenging, and the incidence of conversion to open surgery (COS) is high. This study aimed to review our experience with laparoscopic surgery for diverticular CVF and identify preoperative risk factors for COS.

**Results:**

This was a single institution, retrospective, observational study of 11 patients (10 males and 1 female) who underwent laparoscopic sigmoid colon resection with fistula resection for diverticular CVF from 2014 to 2019. Preoperative magnetic resonance imaging (MRI) was performed to evaluate the fistula location in the bladder, patency of the rectovesical pouch (i.e., the destination of dissection procedure between sigmoid colon and bladder) and estimate the contact area between the sigmoid colon and bladder. The relationship between preoperative variables and COS incidence was analyzed between completed laparoscopy and COS groups. The overall incidence of postoperative morbidity (Clavien–Dindo classification Grade II or higher) was 36% (4/11). Severe morbidity, reoperation, and mortality were not observed. The incidence of COS was 27% (3/11). Posterior bladder fistulas were significantly associated with COS. CVFs located on the posterior bladder appears to be a risk factor for COS. Identifying risk factors for COS preoperatively could help guide the intraoperative course.

## Introduction

Fistulae complicate approximately 20% of colonic diverticulitis cases, which require surgical intervention and most commonly involve the bladder (65–69%) [1]. Diverticular colovesical fistulae (CVF) seldom close spontaneously and cause various sequelae, including multi-organism urinary tract infections (UTIs), pneumaturia, cystitis, pyelonephritis, urinary sepsis, and renal impairment. Therefore, operative management is the recommended treatment for CVF [2–10].

Several retrospective case series have suggested both the safety and feasibility of laparoscopic management of diverticular CVF in a highly selected patient-group. Laparoscopic treatment results in an earlier return of bowel function and shorter hospitalization time with low overall morbidity [9, 11–13]. However, laparoscopic surgery for diverticular CVF is still technically challenging because of extensive inflammation and abscess formation. These complications may explain why the incidence of conversion to open surgery (COS) ranges from 0 to 50% [9, 11–20]. Identification of preoperative risk factors for COS is critical, as evaluating risk can help guide the decision on conversion during the surgery. Knowing risk in advance allows surgeons to determine whether open surgery should be the initial approach, thereby avoiding the potential complications that may lead to intraoperative COS. Furthermore, awareness of these risk factors allows for more thorough briefings of the patients, allowing them to be better-informed before giving consent.

This study aimed to review our experience with laparoscopic surgery for diverticular CVF and to identify preoperative risk factors for COS by comparing completed laparoscopy and COS groups.

## Main text

### Methods

#### Demographics

All patients who underwent elective laparoscopic sigmoid colon resection with fistula resection for diverticular CVF from 2014 to 2019 were identified in our prospective, single-center institutional database. Emergent cases were not included.

All patients underwent a preoperative colonoscopy, computed tomography, and magnetic resonance imaging (MRI) to confirm CVF and eliminate the possibility of colon cancer. Cystoscopy was also performed on all patients to confirm the patency of both ureteral orifices and exclude urological malignancy.

Patient data were collected through electronic medical record systems. Data included information on age, sex, body mass index (BMI), previous abdominal operations, American Society of Anesthesiologists physical status (ASA–PS) classification, and preoperative hematological inflammatory findings including white blood cells (WBCs) and C-reactive protein (CRP).

**Fig. 1:**
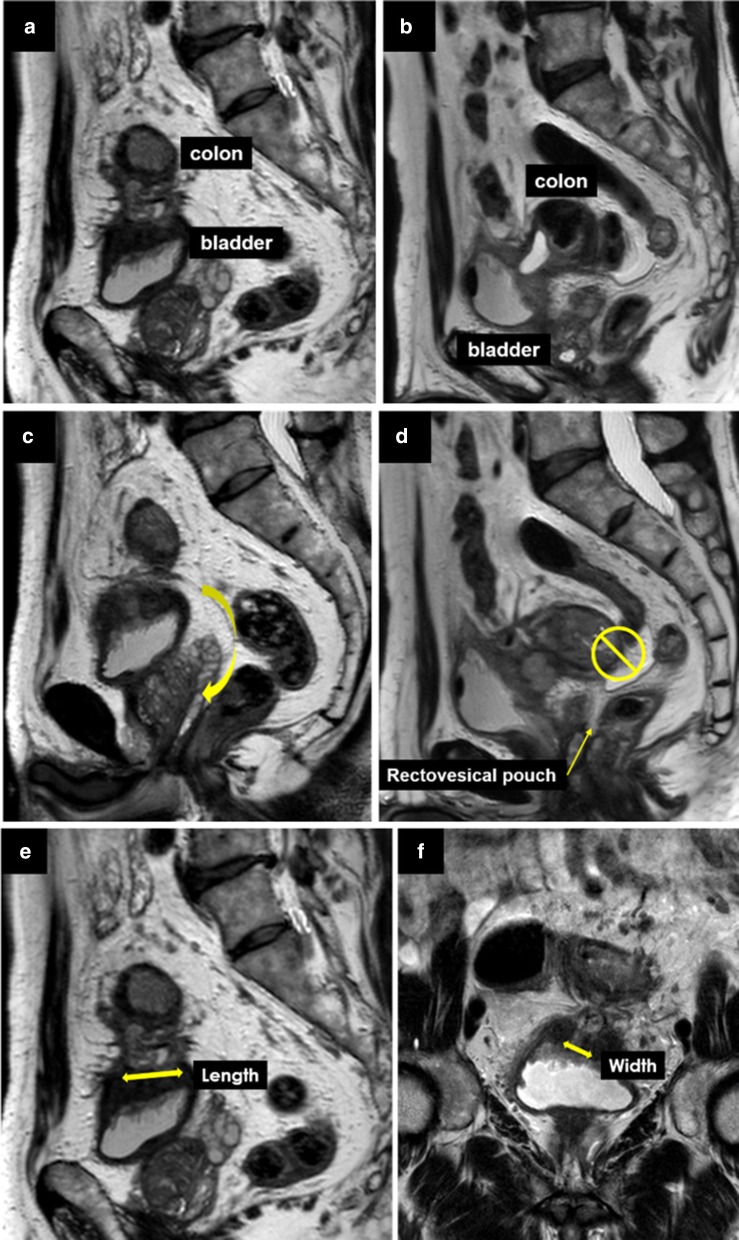
Fistula location on bladder and patency of the rectovesical pouch were evaluated using preoperative magnetic resonance images (MRI) from a representative case, and the estimated contact area between the sigmoid colon and bladder (eCA) was calculated as the product of the length and width between the sigmoid colon and bladder on two-dimensional MRI. **a** Fistula is located on the superior wall of the bladder. **b** Fistula is located in the posterior bladder. **c**: The patency of the rectovesical pouch is preserved and the fistula can be encircled. **d** The rectovesical pouch is closed and cannot be approached directly. **e** Length between the sigmoid colon and bladder on sagittal view. **f** Width between the sigmoid colon and bladder on coronal view

In all cases, the surgical strategy, including the combined resection extent of the bladder wall, was determined based on preoperative MRI findings. MR images were evaluated for the following features: location of the fistula on the bladder, patency of the rectovesical pouch, and estimated contact area (eCA) between the sigmoid colon and bladder. eCA was calculated as the product of the length and width between the sigmoid colon and bladder on two-dimensional MR images (Fig. 1). Intraoperative measurements of interest included operative time, blood loss, rate of positive bladder leak tests, type of bladder repair, stoma creation, complications, and COS. Postoperative measurements of interest included morbidity, timing of Foley catheter removal, length of hospital stay, reoperation, and mortality. The Foley catheter was removed on postoperative day 7 after confirming negative results using cystography; however, the schedule was moved forward if the patients demanded it and was delayed when there was concern about leakage.

Written informed consent was obtained preoperatively from all patients. The protocol for this retrospective study was approved by the ethics committee of the University of Tsukuba Hospital (Registration No. R01–271). The study conforms to the provisions of the Declaration of Helsinki in 1964 (as revised in Brazil in 2013).

#### Operative technique

Laparoscopy was performed using five ports. First, the sigmoid colon was detached from the bladder using electrocautery. The left ureter, gonadal vessels, inferior mesenteric plexus, and superior hypogastric plexus were identified and preserved. In patients with severe inflammation, left or bilateral ureter stents were inserted to facilitate ureter identification. Fistula resection was performed, followed by sigmoid colon resection in a standard manner. Rectal transection was performed with a linear stapler, and the specimen was extracted through the navel port. Anastomoses were performed using a circular stapler introduced per rectum, and a bladder leak test was routinely performed. When the results were positive, the repair of the bladder wall was performed. When the results were negative, it was deemed to be unnecessary. Finally, a pelvic drain was inserted.

#### Statistical analysis

Quantitative data were reported as median (range) and compared using the Mann–Whitney U test. Qualitative data were reported as the number of patients (percentage) using Fisher’s exact test. All tests performed were two-tailed with the level of significance set at p < 0.05. All statistical analyses were conducted using EZR (Saitama Medical Center, Jichi Medical University, Saitama, Japan), a graphical user interface for R (The R Foundation for Statistical Computing, Vienna, Austria). EZR is a modified version of R commander designed for statistical functions frequently used in biostatistics [21]. We used statistics in a descriptive fashion, realizing that with the number of subjects, no robust statistical analysis was possible.

### Results

During the study period, eleven patients underwent laparoscopic sigmoid colon resection with fistula resection for diverticular CVF. The patient characteristics are described in Table 1. The median age of the cohort was 55 years (29–73), and 91% (10/11) of patients were male. The median BMI was 24 kg/m^2^ (20–29 kg/m^2^), and the majority of patients were of ASA–PS class 2 or 3 (total 82%, 9/11). None of the patients underwent previous abdominal operations. Regarding preoperative laboratory findings, the median WBC was 6900/µL (4300–14,000/µL), and the median CRP was 0.64 mg/dL (< 0.03–3.93 mg/dL). The preoperative MRI studies showed that the majority of fistulas were located on the bladder’s superior surface (73%, 8/11) rather than the posterior surface. Patency of the rectovesical pouch was observed in 55% (6/11) of patients, and the median eCA was 450 mm^2^ (100–1575 mm^2^).

**Table 1:** Patient characteristics, intraoperative outcomes, and postoperative outcomes

	N = 11
Age (years)	55 [29–73]^a^
Sex (male)	10 (91%)
BMI (kg/m^2^)	24 [20–29]^a^
ASA-PS	
1	2 (18%)
2	5 (45%)
3	4 (36%)
Prior abdominal operations	0
Preoperative blood exams	
WBC (/µL)	6900 [4300–14,000]^a^
CRP (mg/dL)	0.64 [< 0.03–3.93]^a^
Operative time (min)	251 [207–385]^a^
Blood loss (mL)	100 [0–560]^a^
Bladder leak test (positive)	4 (57%)
Type of bladder repair	
Simple closure	6 (55%)
Partial resection	0
No repair	5 (45%)
Stoma creation	0
Complication	0
Conversion to open surgery	3 (27%)
Overall morbidity	4 (36%)
(Clavien–Dindo grade 2 or more)	
UTI	2
Ileus	1
Rest abscess	1
Timing of Foley catheter removal (POD)	7 [3–11]^a^
Length of hospital stay (days)	13 [8–21]^a^
Reoperation	0
Mortality	0

^a^Median [range]

*BMI* body mass index, *ASA-PS* American Society of Anesthesiologists physical status, *WBC* white blood cell, *CRP*: C-reactive protein, *UTI* urinary tract infection, *POD* postoperative day

#### Intraoperative and postoperative outcomes

The median operative time was 251 min (207–385 min), and median blood loss was 100 mL (0–560 mL). Simple closure of the bladder wall was performed in six patients (55%) with a positive bladder leak test. No intraoperative complications occurred, and no stomas were required. The overall incidence of COS was 27% (3/11 patients) (Table 1).

The overall incidence of postoperative morbidity (Clavien–Dindo classification Grade II or higher) was 36% (4/11 patients). UTIs occurred in two patients, and ileus and pelvic abscess occurred in one each. The median timing of Foley catheter removal was postoperative day 7 (range of 3–11), and the median hospital stay was 13 days (range of 8–21). No reoperations or mortalities occurred (Table 1).

#### Preoperative risk factors for COS

The relationship between each variable and the incidence of COS is summarized in Table 2. Regarding the influence of fistula location, posterior bladder fistulas were significantly associated with COS, while superior fistulas were not (3/3 [100%] vs. 0/8 [0%]). Age, sex, BMI, preoperative laboratory, and the other MRI findings were not associated with COS.

**Table 2:** Relationship between preoperative variables and conversion to open surgery

	N	COS	OR [95% CI]
Age (years)			
< 60	6	1 (17%)	2.97
60 ≦	5	2 (40%)	[0.11–235]
Sex			
Male	10	3 (30%)	0
Female	1	0	[0–104]
BMI (kg/m^2^)			
< 25	7	3 (43%)	0
25 <	4	0	[0–4.16]
WBC (/µL)			
< 8000	6	2 (33%)	0.53
8000 <	5	1 (20%)	[0.007–14.5]
CRP (mg/dL)			
< 1.0	8	3 (38%)	0
1.0 <	3	0	[0–7.13]
Fistula location on bladder			
Superior	8	0	N/A
Posterior	3	3 (100%)	
Patency of rectovesical pouch			
Yes	6	0	N/A
No	5	3 (60%)	
eCA (mm^2^)			
< 500	6	0	N/A
500 ≤	5	3 (60%)	

*COS* conversion to open surgery, *OR* odds ratio, *CI* confidential interval, *BMI* body mass index, *WBC* white blood cell, *CRP* C–reactive protein, *eCA* estimated contact area between sigmoid colon and bladder, *N/A* not available

### Discussion

We reviewed our experience with laparoscopic surgery for diverticular CVF. In our cohort, no severe morbidities or mortalities were observed. The incidence of COS was as high as 27%, and a posterior bladder fistula location was a risk factor.

Previous studies have shown that a laparoscopic colectomy can be safely utilized for complicated diverticulitis; however, most reports were limited by exceedingly small cohorts and highly selected patients [11, 14–16]. Recently, a large study of 111 consecutive diverticular fistula cases with minimal exclusion was reported by Martinolich et al. [22]. Although they did not refer to Clavien-Dindo classification and other diverticular fistulae, including colovaginal, coloenteric, colocutaneous, and colocolonic fistulae, that were included in their cohort, the overall incidence of postoperative complications was 26.4%. In our study, although the overall incidence of postoperative morbidity was as high as 36%, all were no higher than Grade II of the Clavien–Dindo classification for severe complications. Based on these results, it could be concluded that laparoscopic surgery for diverticular CVF is safe and feasible.

Several small retrospective studies on diverticular fistulas have reported that the incidence of COS ranges from 0 to 50%, [9, 11–20], and patients with a preoperative diagnosis of CVF were most likely to require COS. Recent studies on CVF by Badic et al. [14] and Martinolich et al. [22] reported COS incidence rates of 43% and 42%, respectively, comparable to our rate of 27%. During laparoscopic surgery in general, previously reported risk factors for COS included old age, male sex, high BMI, and previous abdominal operations [23–25]. Diverticular fistula cases, in particular, showed severe inflammation or dense fibrosis, impeding safe dissection, or ureteral visualization to be the most frequent reason for COS [22]. In our study, although age, BMI, and previous abdominal operations were not significantly correlated with COS, this may simply be a consequence of the small sample size. We proposed three novel MRI features as preoperative risk factors for COS and finding that fistula location on the bladder appeared to correlate with COS. This study could not demonstrate that the patency of the rectovesical pouch and eCA had a significant correlation with COS because of the small sample size; however, it is considered that COS is not always affected by a risk factor. It will be necessary to identify more cases and further investigate this topic.

As described by Engledowe et al. [12], small fistulas with accompanying inflammation of the bladder wall were not formally closed, and the Foley catheter was left in place for decompression for 5–7 postoperative days. There were no complications related to urinary leakage in these patients. In our study, bladder wall repair was not performed in patients with a negative leak test, and simple closure without partial resection was sufficient, regardless of the leak test outcome. As a result, no postoperative urinary leakages were observed in our cohort.

The ability to identify individual patient’s risk factors of COS can aid surgeons in selecting those who may benefit from primary open surgery, thereby potentially reducing operative time, morbidity, and costs [25]. However, because our results suggested the feasibility of laparoscopic surgery for CVF, it is not necessary to delay the primary laparoscopic approach. The conventional issue was that the decision on COS in the operating room could be subjective and dependent on individual surgeon skills. Just by identifying objective preoperative risk factors associated with COS, it can provide a lower threshold for proceeding with the potentially inevitable open approach and can catalyze the decision on earlier COS.

### Conclusions

Laparoscopic surgery for diverticular CVF was safe, despite the high incidence of COS. In addition, CVFs located on the posterior bladder were risk factors for COS. Finally, understanding the risk factors for COS preoperatively could be important to guide the operative course.

## Limitation

The current study has several limitations. First, it was a retrospective, single-center analysis of clinical records. Second, because the overall sample size was very small, the accuracy of our outcome analyses was limited, and we considered that it is statistically inappropriate to perform a multivariate analysis.

## Data Availability

The datasets used and/or analyzed during the current study are available from the corresponding author on reasonable request.

## Abbreviations

CVF: Colovesical fistula
COS: Conversion to open surgery
MRI: Magnetic resonance imaging
UTI: Urinary tract infection
BMI: Body mass index
ASA-PS: American Society of Anesthesiologists physical status
WBC: White blood cell
CRP: C-reactive protein
eCA: Estimated contact area

## References

[1] Woods RJ, Lavery IC, Fazio VW, Jagelman DG, Weakley FL (1988). Internal fistulas in diverticular disease. Dis Colon Rectum.

[2] Scozzari G, Arezzo A, Morino M (2010). Enterovesical fistulas: diagnosis and management. Tech Coloproctol.

[3] Garcea G, Majid I, Sutton CD, Pattenden CJ, Thomas WM (2006). Diagnosis and management of colovesical fistulae; 6-year experience of 90 consecutive cases. Colorectal Dis.

[4] Daniels IR, Bekdash B, Scott HJ, Marks CG, Donaldson DR (2002). Diagnostic lessons learnt from a series of enterovesical fistulae. Colorectal Dis.

[5] Golabek T, Szymanska A, Szopinski T, Bukowczan J, Furmanek M, Powroznik J (2013). Enterovesical fistulae: aetiology, imaging, and management. Gastroenterol Res Pract..

[6] Kavanagh D, Neary P, Dodd JD, Sheahan KM, O’Donoghue D, Hyland JM (2005). Diagnosis and treatment of enterovesical fistulae. Colorectal Dis.

[7] Lynn ET, Ranasinghe NE, Dallas KB, Divino CM (2012). Management and outcomes of colovesical fistula repair. Am Surg.

[8] De Moya MA, Zacharias N, Osbourne A, Butt MU, Alam HB, King DR (2009). Colovesical fistula repair: is early Foley catheter removal safe?. J Surg Res.

[9] Cirocchi R, Cochetti G, Randolph J, Listorti C, Castellani E, Renzi C (2014). Laparoscopic treatment of colovesical fistulas due to complicated colonic diverticular disease: a systematic review. Tech Coloproctol.

[10] Ferguson GG, Lee EW, Hunt SR, Ridley CH, Brandes SB (2008). Management of the bladder during surgical treatment of enterovesical fistulas from benign bowel disease. J Am Coll Surg.

[11] Cirocchi R, Arezzo A, Renzi C, Cochetti G, D’Andrea V, Fingerhut A (2015). Is laparoscopic surgery the best treatment in fistulas complicating diverticular disease of the sigmoid colon? A systematic review. Int J Surg..

[12] Engledow AH, Pakzad F, Ward NJ, Arulampalam T, Motson RW (2007). Laparoscopic resection of diverticular fistulae: a 10-year experience. Colorectal Dis.

[13] Maciel V, Lujan HJ, Plasencia G, Zeichen M, Mata W, Jorge I (2014). Diverticular disease complicated with colovesical fistula: laparoscopic versus robotic management. Int Surg.

[14] Badic B, Leroux G, Thereaux J, Joumond A, Gancel CH, Bail JP (2017). Colovesical fistula complicating diverticular disease: a 14-year experience. Surg Laparosc Endosc Percutan Tech..

[15] Bhakta A, Tafen M, Glotzer O, Canete J, Chismark AD, Valerian BT (2016). Laparoscopic sigmoid colectomy for complicated diverticulitis is safe: review of 576 consecutive colectomies. Surg Endosc.

[16] Laurent SR, Detroz B, Detry O, Degauque C, Honore P, Meurisse M (2005). Laparoscopic sigmoidectomy for fistulized diverticulitis. Dis Colon Rectum.

[17] Klarenbeek BR, Veenhof AA, Bergamaschi R, van der Peet DL, van den Broek WT, de Lange ES (2009). Laparoscopic sigmoid resection for diverticulitis decreases major morbidity rates: a randomized control trial: short-term results of the Sigma Trial. Ann Surg.

[18] Cirocchi R, Farinella E, Trastulli S, Sciannameo F, Audisio RA (2012). Elective sigmoid colectomy for diverticular disease. Laparoscopic vs open surgery: a systematic review. Colorectal Dis..

[19] Marcucci T, Giannessi S, Giudici F, Riccadonna S, Gori A, Tonelli F (2017). Management of colovesical and colovaginal diverticular fistulas our experience and literature reviewed. Ann Ital Chir.

[20] Tomizawa K, Toda S, Tate T, Hanaoka Y, Moriyama J, Matoba S (2019). Laparoscopic surgery for colovesical fistula associated with sigmoid colon diverticulitis: a review of 39 cases. J Anus Rectum Colon..

[21] Kanda Y (2013). Investigation of the freely available easy-to-use software ‘EZR’ for medical statistics. Bone Marrow Transplant.

[22] Martinolich J, Croasdale DR, Bhakta AS, Ata A, Chismark AD, Valerian BT (2019). Laparoscopic surgery for diverticular fistulas: outcomes of 111 consecutive cases at a single institution. J Gastrointest Surg..

[23] Hu ASY, Menon R, Gunnarsson R, de Costa A (2017). Risk factors for conversion of laparoscopic cholecystectomy to open surgery—A systematic literature review of 30 studies. Am J Surg.

[24] Philip Rothman J, Burcharth J, Pommergaard HC, Viereck S, Rosenberg J (2016). Preoperative risk factors for conversion of laparoscopic cholecystectomy to open surgery—A systematic review and meta-analysis of observational studies. Dig Surg..

[25] Finnerty BM, Wu X, Giambrone GP, Gaber-Baylis LK, Zabih R, Bhat A (2017). Conversion-to-open in laparoscopic appendectomy: a cohort analysis of risk factors and outcomes. Int J Surg..

